# Alcohol-dysregulated miR-30a and miR-934 in head and neck squamous cell carcinoma

**DOI:** 10.1186/s12943-015-0452-8

**Published:** 2015-10-15

**Authors:** Maarouf A. Saad, Selena Z. Kuo, Elham Rahimy, Angela E. Zou, Avinaash Korrapati, Mehran Rahimy, Elizabeth Kim, Hao Zheng, Michael Andrew Yu, Jessica Wang-Rodriguez, Weg M. Ongkeko

**Affiliations:** Division of Otolaryngology-Head and Neck Surgery, Department of Surgery, University of California, La Jolla, San Diego, CA USA; Department of Pathology, Veterans Administration Health Care System, San Diego, CA USA; Department of Pathology, University of California, San Diego, CA USA

**Keywords:** Head and neck squamous cell carcinoma, microRNA, RNA-sequencing, Alcohol, Acetaldehyde

## Abstract

**Background:**

Alcohol consumption is a well-established risk factor for head and neck squamous cell carcinoma (HNSCC); however, the molecular mechanisms by which alcohol promotes HNSCC pathogenesis and progression remain poorly understood. Our study sought to identify microRNAs that are dysregulated in alcohol-associated HNSCC and investigate their contribution to the malignant phenotype.

**Method:**

Using RNA-sequencing data from 136 HNSCC patients, we compared the expression levels of 1,046 microRNAs between drinking and non-drinking cohorts. Dysregulated microRNAs were verified by qRT-PCR in normal oral keratinocytes treated with biologically relevant doses of ethanol and acetaldehyde. The most promising microRNA candidates were investigated for their effects on cellular proliferation and invasion, sensitivity to cisplatin, and expression of cancer stem cell genes. Finally, putative target genes were identified and evaluated *in vitro* to further establish roles for these miRNAs in alcohol-associated HNSCC.

**Results:**

From RNA-sequencing analysis we identified 8 miRNAs to be significantly upregulated in alcohol-associated HNSCCs. qRT-PCR experiments determined that among these candidates, *miR-30a* and *miR-934* were the most highly upregulated *in vitro* by alcohol and acetaldehyde. Overexpression of *miR-30a* and *miR-934* in normal and HNSCC cell lines produced up to a 2-fold increase in cellular proliferation, as well as induction of the anti-apoptotic gene *BCL-2*. Upon inhibition of these miRNAs, HNSCC cell lines exhibited increased sensitivity to cisplatin and reduced matrigel invasion. miRNA knockdown also indicated direct targeting of several tumor suppressor genes by *miR-30a* and *miR-934*.

**Conclusions:**

Alcohol induces the dysregulation of *miR-30a* and *miR-934*, which may play crucial roles in HNSCC pathogenesis and progression. Future investigation of the alcohol-mediated pathways effecting these transformations will prove valuable for furthering the understanding and treatment of alcohol-associated HNSCC.

**Electronic supplementary material:**

The online version of this article (doi:10.1186/s12943-015-0452-8) contains supplementary material, which is available to authorized users.

## Background

Based on incidence and mortality, head and neck squamous cell carcinoma (HNSCC) is the 6^th^ leading cancer worldwide with an estimated 900,000 newly diagnosed cases each year [1]. The major risk factors for HNSCC include tobacco and alcohol, which account for at least 75 % of all cases diagnosed in Europe, the United States, and other industrialized regions [2]. Tobacco use and alcohol consumption are two habits strongly linked with each other that have been shown to act synergistically in HNSCC, but many studies have also demonstrated each substance to be an independent risk factor [2–5]. Despite this knowledge, there is little evidence that people have modified their alcohol intake, as alcohol consumption has remained high throughout the years whereas tobacco usage has steadily declined [3, 4]. Meanwhile, a molecular understanding of the pathogenesis of alcohol-related HNSCC remains elusive and poorly understood. Since ethanol alone is not considered a carcinogen, most studies involving alcohol and cancer focus on its ability to increase penetration of carcinogens, interfere with DNA repair mechanisms, or cause DNA damage through acetaldehyde, the first metabolite of ethanol and an established carcinogen [6, 7].

Recently, numerous studies have revealed that microRNAs (miRNAs) play a role in the initiation and progression of many cancers, including HNSCC. miRNAs are a class of non-coding RNAs 19–25 nucleotides in length that can regulate gene expression by binding to the partially complementary regions of messenger RNA targets. It is proposed that miRNAs regulate up to a third of protein coding genes, in turn impacting myriad cellular processes including cellular growth, differentiation, self-renewal, apoptosis, survival, and migration [8, 9]. Analyzing the expression of miRNAs in cancer compared to normal tissue and investigating the functional significance of their dysregulation may be pivotal for improving early diagnosis, predicting prognosis, and establishing specific therapeutics. Several studies have previously revealed aberrations in miRNA expression in HNSCC tumors [10–12], but conclusive biomarkers have yet to be established [8]. Furthermore, there is little evidence about how miRNA dysregulation relates to many HNSCC clinical risk factors, which may prove vital for a clearer molecular understanding of how these factors play a role in carcinogenesis. One previous study analyzed the association between four pre-selected miRNAs and certain clinical features and risk factors, including alcohol consumption. Using multivariate comparisons, it found that *miR-375* was associated with tumor site, stage, and alcohol consumption in HNSCC [13]. Our study aimed to expand current understanding of the link between alcohol consumption and miRNA expression by using next-generation RNA-sequencing data from 136 HNSCC patients to identify differentially expressed candidates among 1,046 annotated miRNAs. We subsequently investigated the alcohol-associated miRNAs in normal oral keratinocyte cell cultures in order to establish if these miRNAs may be crucial for their malignant transformation. After selecting the miRNAs that were the most dysregulated by alcohol and acetaldehyde, we evaluated how modulation of their expression would induce changes in cellular proliferation, sensitivity to cisplatin, and invasion, and finally sought to identify their mRNA targets The results of this study demonstrate that alcohol consumption regulates several miRNAs that likely play significant roles in the alcohol-associated carcinogenesis of HNSCC.

## Results

### Identification of microRNAs associated with alcohol consumption in HNSCC

**Fig. 1:**
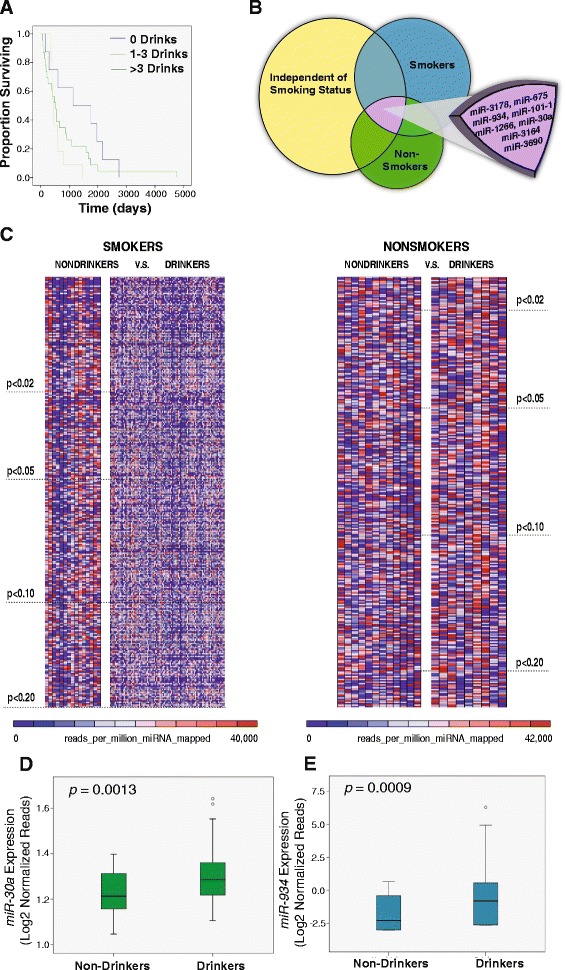
RNA-sequencing analysis identifies 8 microRNAs that are associated with alcohol consumption. (**a**) Kaplan-Meier analysis indicates that increased alcohol consumption leads to poorer patient survival in our patient population (*n* = 42). (**b**) Venn diagram schematic conveying our approach to determine the 8 alcohol-associated miRNAs. Each circle represents a comparison of the drinkers versus non-drinkers in each of the categories (smokers, non-smokers, or inclusion of all patients). (**c**) Heatmaps represent the reads per million miRNA mapped (RPKM) of the most statistically significant dysregulated miRNAs between drinkers and non-drinkers. miRNAs are listed by order of p-value with the most significant located towards the top. (**d**) Log-transformed *miR-30a* and (**e**) *miR-934* expression levels in drinkers versus non-drinkers. The middle bar represents the mean, the top and bottom borders represent the 25^th^ and 75^th^ percentile, and the whiskers reach the 5^th^ and 95^th^ percentile. The circles represent outliers

From the TCGA database, we selected 136 HNSCC patients with miRNA-sequencing data providing normalized expression values for 1,046 miRNAs, as well as clinical data documenting both alcohol consumption (drinks per day) and smoking status (pack-years smoked). Using a Kaplan-Meier survival analysis, we were able to verify that our patient cohort demonstrated the risk of alcohol, with patients who consumed alcohol experiencing significantly lower survival rates (Fig. 1a). To account for the two major risk factors of HNSCC, we sorted our patient cohort into 4 groups: (1) nonsmoking non-drinkers, (2) nonsmoking drinkers, (3) smoking non-drinkers, and (4) smoking drinkers (Table 1). In order to identify miRNAs that were dysregulated due to alcohol consumption alone, we compared miRNA expression in drinkers and non-drinkers within both the smoking and non-smoking cohorts. Additionally, we compared miRNA expression and drinkers and non-drinkers using the entire patient population, regardless of smoking status (Fig. 1b-c). From our analysis, we identified 8 miRNAs that were significantly upregulated (*p* < 0.05) in alcohol consumers compared to non-drinkers in all three of our comparisons (Fig. 1d-e, Table 2).

**Table 1:** Summary of patient demographics and clinicopathological characteristics (*n* = 136)

		Non–Drinker (27)		Drinker (109)	
	All No. (%)	Smoker No. (%)	Non–Smoker No. (%)	Smoker No. (%)	Non–Smoker No. (%)
*Gender*					
Male	109 (80 %)	9 (60 %)	8 (67 %)	86 (86 %)	7 (78 %)
Female	27 (20 %)	6 (40 %)	4 (34 %)	14 (14 %)	2 (22 %)
*Lifetime pack–years smoked* ^a^					
None	21 (15 %)	0 (0 %)		0 (0 %)	
>0–40	40 (29 %)	7 (47 %)	N/A	32 (32 %)	N/A
>40	49 (36 %)	4 (27 %)		44 (44 %)	
*Drinks per day*					
None	27 (20 %)			0 (0 %)	0 (0 %)
0–2	25 (18 %)	N/A	N/A	20 (20 %)	5 (56 %)
>2	84 (62 %)			80 (80 %)	4 (44 %)
*Vital Status*					
Deceased	42 (31 %)	6 (40 %)	2 (17 %)	33 (33 %)	1 (11 %)
Living	94 (69 %)	9 (60 %)	10 (83 %)	67 (67 %)	8 (89 %)
*Tumor Site*					
Oral	92 (68 %)	12 (80 %)	9 (75 %)	65 (65 %)	6 (67 %)
Pharyngeal	15 (11 %)	0 (0 %)	3 (25 %)	9 (9 %)	3 (33 %)
Laryngeal	29 (21 %)	3 (20 %)	0 (0%)	26 (26 %)	0 (0 %)
*Stage* ^b^					
Low (I, II)	28 (25 %)	2 (13 %)	2 (29 %)	23 (28%)	1 (14 %)
High (III, IV)	84 (75 %)	13 (87 %)	5 (71 %)	60 (72%)	6 (86 %)
*Grade*					
GX	4 (3 %)	0 (0 %)	0 (0 %)	3 (3 %)	1 (11 %)
G1–G2	92 (68 %)	13 (87 %)	9 (75 %)	65 (65 %)	5 (56 %)
G3–G4	40 (29 %)	2 (13 %)	3 (25 %)	32 (32 %)	3 (33 %)
Total:	136	15	12	100	9

^a^26 patient data not available

^b^24 patient data not available

**Table 2:** List of 8 identified microRNAs and their corresponding fold changes and p-values

miRNA ID	Fold change (Drinkers/Nondrinkers)	*P*-value	Comparison
*hsa-mir-3178*	7.59	0.0398	Drinkers Vs. Nondrinkers (Non-smokers)
	5.18	0.0004	Drinkers Vs. Nondrinkers (Smokers)
	5.77	1.4659E-05	Drinkers Vs. Nondrinkers (Overall)
*hsa-mir-675*	3.57	0.0221	Drinkers Vs. Nondrinkers (Non-smokers)
	8.07	0.0012	Drinkers Vs. Nondrinkers (Smokers)
	5.48	0.0009	Drinkers Vs. Nondrinkers (Overall)
*hsa-mir-934*	5.99	0.0164	Drinkers Vs. Nondrinkers (Non-smokers)
	4.19	0.0237	Drinkers Vs. Nondrinkers (Smokers)
	4.74	0.0122	Drinkers Vs. Nondrinkers (Overall)
*hsa-mir-101*	2.31	0.0491	Drinkers Vs. Nondrinkers (Non-smokers)
	1.25	0.0337	Drinkers Vs. Nondrinkers (Smokers)
	1.76	0.0042	Drinkers Vs. Nondrinkers (Overall)
*hsa-mir-1266*	3.82	0.0163	Drinkers Vs. Nondrinkers (Non-smokers)
	1.53	0.0322	Drinkers Vs. Nondrinkers (Smokers)
	2.60	0.0018	Drinkers Vs. Nondrinkers (Overall)
*hsa-mir-30a*	1.65	0.0383	Drinkers Vs. Nondrinkers (Non-smokers)
	1.67	0.0052	Drinkers Vs. Nondrinkers (Smokers)
	1.66	0.0013	Drinkers Vs. Nondrinkers (Overall)
*hsa-mir-3164*	21.12	0.0469	Drinkers Vs. Nondrinkers (Non-smokers)
	6.71	0.0208	Drinkers Vs. Nondrinkers (Smokers)
	1.94	0.0375	Drinkers Vs. Nondrinkers (Overall)
*hsa-mir-3690*	2.79	0.0429	Drinkers Vs. Nondrinkers (Non-smokers)
	1.63	0.0349	Drinkers Vs. Nondrinkers (Smokers)
	2.12	0.0026	Drinkers Vs. Nondrinkers (Overall)

### *In vitro* ethanol treatment validates alcohol-induced microRNA dysregulation

**Fig. 2:**
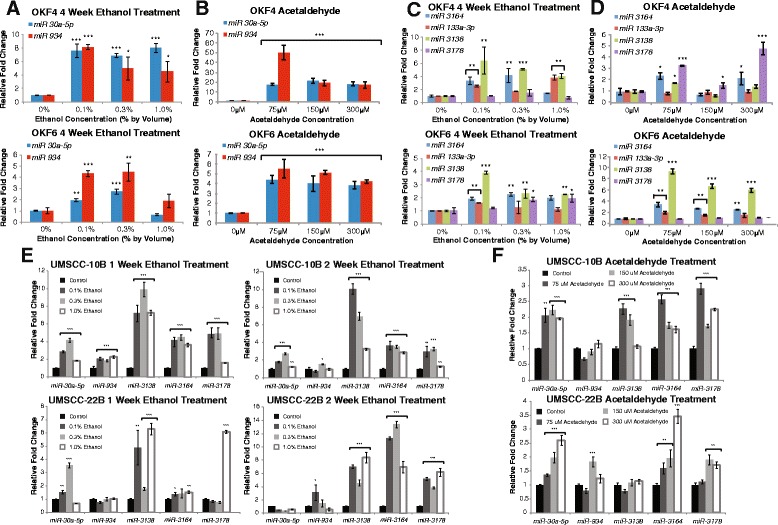
Ethanol and acetaldehyde treatment in normal culture cells verifies clinical data analysis of alcohol-dysregulated microRNAs. (**a**) qRT-PCR demonstrates that long-term ethanol treatment of normal oral epithelial cells upregulates *miR-30a* and *miR-934* in OKF4 and OKF6. (**b**) qRT-PCR demonstrates that 48-hour acetaldehyde treatment of normal oral epithelial cells also upregulates *miR-30a* and *miR-934* in OKF4 and OKF6. (**c**) Long-term ethanol treatment also upregulates 4 other alcohol-associated miRNAs in OKF4 and OKF6. (**d**) Acetaldehyde treatment also upregulates 4 other alcohol-associated miRNAs in OKF4 and OKF6. (**e**) 1- and 2-week exposure of UMSCC-10B and UMSCC-22B results in upregulation of the candidate miRNAs, as does (**f**) 48-hour acetaldehyde treatment of the two HNSCC cell lines. All data is presented as the mean and error bars represent standard deviation. *p< 0.05, **p<0.01, ***p<0.001, Student’s *t*-test

In order to verify that the miRNAs we identified were associated with alcohol consumption, we performed *in vitro* ethanol treatment using early passage oral epithelial culture cells OKF4 and OKF6. We used normal oral epithelial cells in order to address whether alcohol directly promotes dysregulation of our identified miRNAs as an early event in malignant transformation. We first assessed the viability of our cell lines under a large range of ethanol doses (0-10 % by volume) over 48 h using an MTS assay and found that there was no significant toxicity up to 1 % (Additional file 1: Figure S1). In order to mimic short- and long-term exposure to alcohol, we treated our cell cultures with biologically relevant concentrations of ethanol for a period of 1, 2, or 4 weeks, using a range of doses resembling the blood alcohol levels attained by social drinkers to heavy drinkers, 0.1 % (17 mM) and 0.3 % (51 mM) by volume, respectively, with 1 % (51 mM) ethanol serving as the upper limit control (see Methods). Of the eight miRNAs that we identified from the clinical data, *miR-30a-5p, miR-934, miR-3164*, and *miR-3178* were upregulated from long-term (4-week) ethanol exposure in both OKF4 and OKF6 (Figs. 2a and c). Additionally, two other miRNAs, *miR-133a* and *miR-3138*, were upregulated in the alcohol-consuming patients in two categories and also verified *in vitro* (Fig. 2c). We next sought to investigate what duration of ethanol exposure in the normal cells was sufficient to induce overexpression of these miRNAs. While 1-week ethanol exposure resulted in the upregulation of *miR-934* and *miR-3178* in OKF4 (Additional file 1: Figure S1), we found that 4-week ethanol exposure was necessary in order to generate consistent upregulation of the miRNAs of interest. In addition, we exposed the established HNSCC cell lines UMSCC-10B and UMSCC-22B to ethanol in order to assess the dysregulation of our candidate miRNAs in cancer cells. Shorter-term 1- and 2-week ethanol treatments were both sufficient to result in overexpression of *miR-30a*, *miR-934*, *miR-3138*, *miR-3164*, and *miR-3178* (Fig. 2e).

### *In vitro* acetaldehyde treatment further validates alcohol-induced microRNA dysregulation

Although alcohol consumption has been determined to be an independent risk factor for HNSCC, the precise mechanism by which alcohol exerts this effect remains unclear, since ethanol itself is not carcinogenic. It has been postulated that acetaldehyde, the first metabolite of ethanol, contributes to alcohol-associated cancer risk, as it has been shown to produce mutagenic effects such as chromosomal aberrations and DNA cross-links [6]. While the bulk of alcohol metabolism is carried out in the liver, it may also take place via intracellular enzymes in the esophageal and oral mucosa [7] . Furthermore, acetaldehyde is also formed in high concentrations in saliva by the oral microflora [14] . In order to further verify that our selected miRNAs play a role in alcohol-related pathogenesis, we used physiologically relevant doses of acetaldehyde as determined by saliva concentrations in alcohol consumers [14], ranging from 75 μM to 300 μM to represent light to heavy drinking habits. A 24 h MTS assay was performed to test a large range of acetaldehyde doses and their effects on cellular viability. We show that at the clinically relevant doses, there is an inconsequential difference in cellular proliferation (Additional file 1: Figure S1). Upon exposure to acetaldehyde treatment, the normal epithelial cells showed a similar increase in expression of the same 6 miRNAs as the long-term ethanol treatment (Fig. 2b and d). Meanwhile, acetaldehyde-exposed UMSCC-10B and UMSCC-22B similarly overexpressed *miR-30a, miR-934*, *miR-3138*, *miR-3164*, and *miR-3178* (Fig. 2f). Taken together, our *in vitro* data suggests that the dysregulation of these miRNAs are associated with alcohol consumption and metabolism, and may even play an early role in the malignant transformation of normal oral cells.

### Overexpression of miR-30a and miR-934 induces the expression of *BCL-2* and increases cellular proliferation

**Fig. 3:**
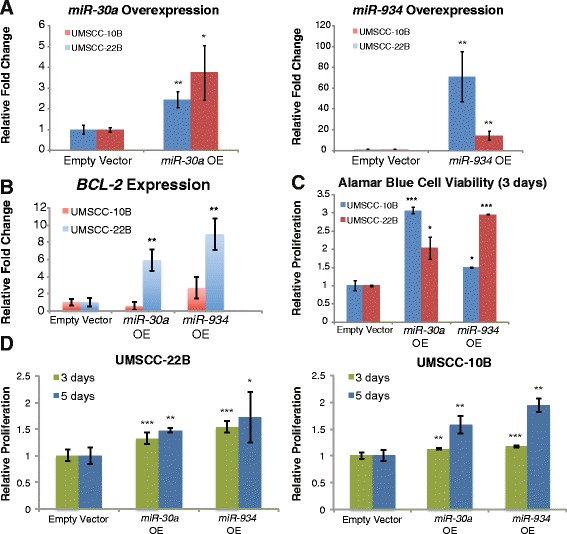
Overexpression of *miR-30a* and *miR-934* induces the expression of cancer stem cell genes and increases cellular proliferation in HNSCC cell lines. (**a**) Transient transfection of miRNA expression plasmids is able to overexpress both miRNAs in the two HNSCC cell lines UMSCC-10B and UMSCC-22B. (**b**) Overexpression of *miR-30a* and *miR-934* resulted in the increased expression of anti-apoptotic gene *BCL-2*. Enforced expression of both *miR-30a* and *miR-934* promotes increased significantly cellular proliferation in HNSCC cell lines as demonstrated by (**c**) Alamar blue assays and (**d**) MTS assays. Double transfections were performed for the 5-day experiments. All data is presented as the mean and error bars represent standard deviation. *p<0.05, **p<0.01, ***p<0.001, Student’s *t*-test

To functionally characterize the effects of our identified alcohol-regulated miRNAs, we investigated their influence on cellular proliferation as well as their relationship with established cancer genes, including the anti-apoptotic gene *BCL-2*. We selected *miR-30a-5p* and *miR-934* for functional characterization, as they were the most consistently dysregulated in the clinical data, ethanol treatments, and acetaldehyde treatments. Experiments were performed in the HNSCC cell lines UMSCC-10B and UMSCC-22B as proof of principle, since the normal early passage cell cultures are highly sensitive to transfection reagents. Transfection efficiency for all experiments was greater than 70 % and the overexpression of each miRNA, in each cell line, was measured by qRT-PCR (Fig. 3a). Overexpression of *miR-30a* and *miR-934* increased the expression of *BCL-2* in both UMSCC-10B and UMSCC-22B (Fig. 3b). To test effects on proliferation, an Alamar blue assay and an MTS assay were each performed on *miR-30a-5p* and *miR-934*-transfected UMSCC-10B and UMSCC-22B. Both miRNAs promoted increased proliferation, with with *miR-30a* and *miR-934* inducing nearly 3-fold increases over an experiment period of 3 days in UMSCC-22B (Fig. 3c-d).

### Inhibition of miR-30a and miR-934 sensitizes cells to cisplatin and decreases cellular invasion

**Fig. 4:**
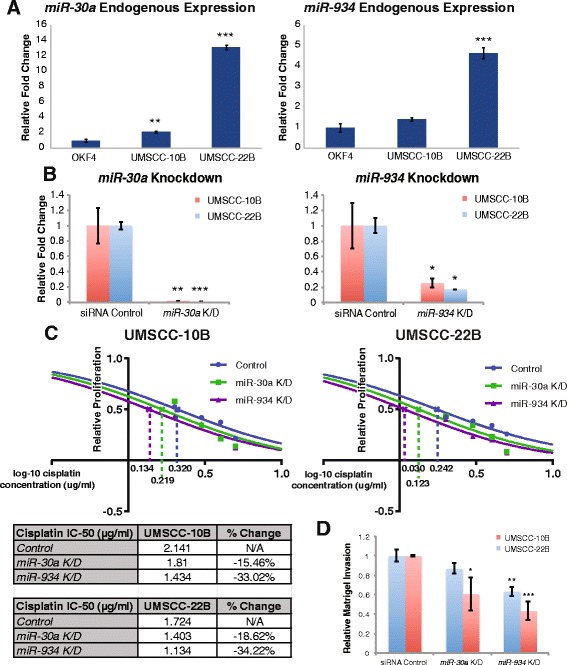
Inhibition of *miR-30a* and *miR-934* decreases cancer stem cell genes, enhances cisplatin-induced cell death, and decreases invasive abilities in HNSCC cell lines. (**a**) qRT-PCR demonstrates that *miR-30a* and *miR-934* are significantly upregulated in HNSCC cell lines, UMSCC-10B and UMSCC-22B, compared to the normal cell line, OKF4. (**b**) Knockdown of baseline expression in HNSCC cell lines was measured by qRT-PCR, resulting in75% knockdown or greater in the cell lines for both miRNAs. (**c**) MTS assay depicts that the knockdown of *miR-30a* and *miR-934* sensitizes the HNSCC cell lines to cisplatin as shown by the reduction in cisplatin IC-50. IC-50 values were obtained by fitting a dose–response curve to the averaged values for relative proliferation under each condition and cisplatin dose. (**d**) Matrigel invasion assay shows a decrease in the number of invading cells after knockdown of *miR-30a* and *miR-934* relative to control. All data is presented as the mean and error bars represent standard deviation. *p<0.05, **p<0.01, ***p<0.001, Student’s *t*-test

Since endogenous expression of *miR-30a* and *miR-934* was higher in HNSCC cell lines compared to normal cell lines (Fig. 4a), we inhibited both miRNAs in the HNSCC cell lines UMSCC-10B and UMSCC-22B to further characterize their functional effects. Knockdown of both miRNAs was performed with anti-miRs and produced at least a 75 % reduction in miRNA expression (Fig. 4b). We first performed an MTS assay to test the effects of cisplatin on *miR-30a-* and *miR-934-*inhibited UMSCC-10B and UMSCC-22B. Our results show that knockdown of the miRNAs sensitizes the cells to cisplatin-mediated cell death, even though inhibition of the miRNAs alone had a limited effect on cellular proliferation (Fig. 4c). Additionally, matrigel invasion assays were performed to evaluate the roles of these miRNAs in regulating cellular invasion. Inhibition of *miR-934* resulted in up to 56 % reduction in invasive abilities, while inhibition of *miR-30a* caused up to 40 % reduction (Fig. 4d).

### miR-30a and miR-934 regulate expression of predicted target tumor suppressor genes

**Fig. 5:**
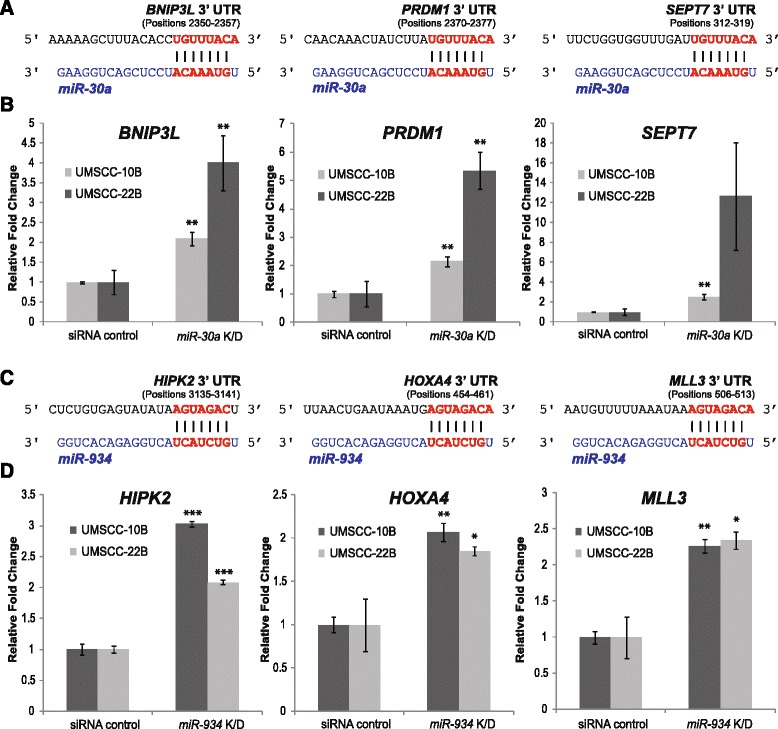
*miR-30a* and *miR-934* knockdown in HNSCC cell lines results in the upregulation of predicted tumor suppressor target genes. (**a**) Putative sequences targeted by *miR-30a* within the 3’ UTR of *BNIP3L, PRDM1*, and *SEPT7*. (**b**) The three predicted targets are significantly upregulated in the UMSCC-10B and UMSCC-22B cell lines following inhibition of *miR-30a* (48 h). (**c**) Putative sequences targeted by *miR-934* within the 3’ UTR of *HOXA4, HIPK2,* and *MLL3.* (**d**) The three predicted targets exhibit increased expression in UMSCC-10B and UMSCC-22B 48 h after knockdown of *miR-934*. All data is presented as the mean and error bars represent standard deviation. *p<0.05, **p<0.01, ***p<0.001, Student’s *t*-test

To further define molecular roles for *miR-30a* and *miR-934* in HNSCC, we identified candidate mRNA targets for both miRNAs using two target prediction algorithms. The tumor suppressor genes *BNIP3L, PRDM9,* and *SEPT7* were each found to have direct 3’UTR binding sites with *miR-30a-5p* (Fig. 5a). Meanwhile, tumor suppressors *HIPK2, HOXA4,* and *MLL3* were predicted to be similarly targeted by *miR-934* (Fig. 5c). Next, we evaluated the expression levels of these putative targets following knockdown of *miR-30a* and *miR-934* in UMSCC-10B and UMSCC-22B. Inhibition of *miR-30a* induced significant upregulation of *BNIP3L, PRDM9,* and *SEPT7* (Fig. 5b), while inhibition of *miR-934* increased the expression of *HIPK2, HOXA4,* and *MLL3* (Fig. 5c), suggesting that the miRNAs exert negative regulatory effects on these established tumor suppressors.

## Discussion

Although alcohol consumption has been identified as a potent risk factor for HNSCC, knowledge regarding its functional role as an independent etiological agent for the disease remains significantly limited. Multiple studies have proposed that the action of alcohol in HNSCC pathogenesis is mediated by its first metabolite acetaldehyde, a well-established carcinogen [15], but others have postulated that alcohol may exert adverse effects directly, either by increasing the cellular uptake and potency of carcinogens such as tobacco [16], or by inducing DNA hypomethylation [17]. The relevance of these proposed models to alcohol-associated HNSCC and the molecular mechanisms by which they may occur have remained obscure; previous investigations of the effects of environmental factors on genetic and epigenetic alterations in HNSCC have largely focused on characterizing tobacco, HPV exposure, or tobacco in association with alcohol [1, 18, 19].

We are the first to globally profile alcohol-induced miRNA dysregulation in HNSCC and to functionally characterize the roles that these miRNAs may play in HNSCC pathogenesis and progression. Through RNA-sequencing analysis of miRNA expression in 136 patients, we identified a panel of 8 miRNAs (*miR-3178*, *miR-675*, *miR-934*, *miR-101*, *miR-1266*, *miR-30a*, *miR-3164*, and *miR-3690*) exhibiting significant upregulation in alcohol-associated HNSCCs, suggesting their role as oncomirs. By subdividing our patient cohort into smokers and non-smokers, and comparing the drinking and non-drinking populations within both groups for unifying miRNA aberrations, we ensured that these miRNA candidates were dysregulated due to alcohol consumption alone, removing the synergistic and likely confounding influence of tobacco.

None of the 8 miRNAs have been previously identified in studies of tobacco or HPV-induced miRNA dysregulation in HNSCC [1]. Furthermore, *miR-3164* and *miR-3690* demonstrate no previous connections to cancer, establishing them as potentially fruitful targets for future investigation. Such findings underscore existing evidence of considerable heterogeneity in the genetic and transcriptional landscapes of HNSCCs associated with different etiologies [1, 18], and pose implications for future distinctions in the diagnostic strategies and clinical management of alcohol-associated HNSCC.

Interestingly, previous studies of *miR-1266* and *miR-3678* characterize their roles as tumor suppressor miRNAs in gastric cancer. *miR-1266* was downregulated in gastric cancer tissues and was shown to inhibit tumor growth and invasion by targeting telomerase reverse transcriptase (hTERT) [20], while *miR-3678* was found to be significantly downregulated in lymphatic metastases [21]. Meanwhile, there exists conflicting evidence regarding the roles of *miR-675* and *miR-101* in various malignancies. *miR-675*, along with its precursor long non-coding RNA *H19,* was found to inhibit invasion and metastasis in hepatocellular carcinomas [22], but was shown to promote the pathogenesis and metastasis of gastric cancer and target the tumor suppressor retinoblastoma in colorectal cancer [23, 24]. Similarly, studies in a number of cancers have found *miR-101* to function as a tumor suppressor by targeting *EZH2* [24, 25], yet it was also implicated as a promoter of growth in estrogen receptor (ER)-positive breast tumors by inducing the activation of Akt [25]. While further investigation of these discrepancies may be necessary, there is mounting evidence that miRNAs may assume tissue-specific or cancer-specific targets and functions via distinct pathways [26, 27]. Therefore, future characterizations of the miRNAs we have identified may find that they indeed exert unique effects in the contexts of HNSCC and alcohol-induced dysregulation.

We found *miR-30a*, *miR-934*, *miR-3164*, and *miR-3178* to be consistently upregulated in oral keratinocytes and HNSCC cell lines following treatments with ethanol and acetaldehyde, demonstrating that dysregulation of these miRNAs is directly mediated by exposure to alcohol and is likely involved in both the early stages of malignant transformation and the progression to increased malignancy in HNSCC. Notably, acetaldehyde alone also induced aberrant expression of the miRNAs, implying that its carcinogenic properties are not only the result of DNA cross-links and chromosomal aberrations as previously characterized [6], but also due to its modulation of miRNA-mediated pathways. These findings also confirm that ethanol-to-acetaldehyde metabolism is responsible, at least in part, for alcohol-related HNSCC pathogenesis. Interestingly, however, the 4 miRNAs failed to exhibit proportional increases in expression in response to increased ethanol or acetaldehyde dosage, with strongest upregulation generally occurring at the lowest ethanol (0.1 %) and acetaldehyde (75 μM) concentrations. This may simply indicate that alcohol-mediated miRNA dysregulation is governed by factors other than intake levels; a previous study of the relationship between carcinogen exposure and homozygous deletion of *p16*^*INK4A*^ in HNSCCs found that the duration, rather than intensity, of tobacco and alcohol use significantly predicted deletion frequency [28]. Further investigation of the relationship between length of alcohol consumption and miRNA expression, in conjunction with the *in vitro* experiments presented here simulating long-term alcohol exposure, may therefore be valuable in characterizing more precisely the conditions under which alcohol-induced miRNA dysregulation occurs.

In particular, we show that enforced expression of *miR-30a* and *miR-934* in HNSCC cell lines promotes the induction of anti-apoptotic gene *BCL-2*, as well as increased cellular proliferation in the HNSCC cell lines. Conversely, inhibition of the miRNAs in HNSCC resulted in reduced invasion, as well as decreased resistance to cisplatin. The potential of these alcohol-dysregulated miRNAs to confer proliferative and invasive properties to HNSCC cells may significantly account for the elevated risks of developing HNSCC and experiencing tumor recurrence that have been observed in drinkers [2–5]. Furthermore, the ability of miRNA suppression to sensitize cells to cisplatin and decrease invasive capabilities establishes *miR-30a* and *miR-934* as potential targets of future therapies specifically tailored to alcohol-related HNSCC. Our findings are consistent with previous studies reporting the ability of *miR-934* and *miR-30a* to promote metastasis as well: *miR-934*, along with host gene *VGLL1*, was found to be upregulated in ER-negative *BRCA1*-positive breast cancers and was associated with the acquisition and maintenance of luminal progenitor characteristics [29]; similarly, *miR-30a* was shown to promote invasion in nasopharyngeal carcinoma (NPC) via the repression of E-cadherin and induction of epithelial-to-mesenchymal transition (EMT) [30].

The putative target genes we have shown to be modulated by both miRNAs further corroborate their proposed roles in HNSCC. *BNIP3L,* potential target of *miR-30a,* is a proapoptotic gene activated by p53 under hypoxic conditions [31, 32]. Meanwhile, *SEPT7* is a cell cycle regulator involved in kinetochore localization and apoptosis [33, 34], and *PRDM1* is a transcriptional repressor and inhibitor of the Wnt signaling pathway [35], which has been shown to be centrally associated with stemness and invasiveness in HNSCCs [36].

Both *SEPT7* and *PRDM1* have been found to be targeted by *miR-30a* in gliomas [35, 37]. The proposed targets for *miR-934* are also potent tumor suppressors: *HOXA4* has been shown to inhibit cell motility and invasion in ovarian carcinoma [38]; *MLL3* is involved in a p53 coactivator complex, with knockout resulting in epithelial tumor formation in mice [39]; and *HIPK2* mediates apoptosis and activates p53 via phosphorylation at Ser46 [40].

## Conclusions

Taken together, our findings indicate a novel interplay between alcohol-mediated dysregulation of miRNAs and key tumorigenic and metastatic processes in HNSCC. Specifically, we have shown that *miR-30a* and *miR-*934 are upregulated in HNSCC patients who are drinkers and that *miR-30a* and *miR-934* expression in HNSCC cell lines produced changes in cellular proliferation and invasion, as well as differential targeting of key tumor suppressors. Studies further evaluating *miR-30a* and *miR-934* in the context of alcohol-induced EMT, along with investigations of other roles of ethanol and acetaldehyde in EMT-associated pathways, may substantially enhance our molecular understanding of alcohol-associated HNSCC and catalyze the development of targeted diagnostics and therapies for the disease.

## Methods

### miRNA-sequencing datasets and differential expression analysis

Level 3-normalized miRNA expression datasets and clinical data for 136 HNSCC patients were obtained from The Cancer Genome Atlas (TCGA) (https://tcga-data.nci.nih.gov/tcga).These patients were separated into four cohorts based on reported alcohol and tobacco use, in order to minimize the confounding influence of smoking on HNSCC prevalence and pathogenesis: (1) nonsmoking non-drinkers, (2) nonsmoking drinkers, (3) smoking non-drinkers, and (4) smoking drinkers (Table 1). Statistical analyses were performed using IBM SPSS Statistics v.22. To account for unequal variance, two-tailed Welch’s t-tests were used for the differential expression analyses. Only p-values below 0.05 were considered statistically significant.

### Survival analysis

42 HNSCC patients in the TCGA database had available data for both alcohol consumption and length of survival. All of these patients were smokers and were subdivided into non-drinkers, light drinkers (1–3 drinks per day) and heavy drinkers (more than 3 drinks per day). Kaplan-Meier plots were graphed using IBM SPSS Statistics v.22 and a log-rank test was used to calculate the *p*-value. Only p-values <0.05 were considered statistically significant.

### Cell culture and treatment with ethanol and acetaldehyde

Normal, early passage, oral epithelial cell linesOKF4 and OKF6 (derived from the floor of the mouth) were gifts from the Rheinwald Lab at Harvard Medical School. The cells were cultured in keratinocyte serum-free media (Life Technologies) supplemented with EGF, bovine pituitary extract, 2 % L-glutamine, 2 % penicillin/streptomycin, and CaCl_2_. These cells were either exposed to ethanol for 1 week or 4 weeks, or to acetaldehyde (Sigma Aldrich) for 48 h.

The HNSCC cell lines UMSCC-10B and UMSCC-22B (derived from laryngeal and hypopharyngeal tumors, respectively) were gifts from Dr. Tom Carey, University of Michigan. The cells were cultured in DMEM supplemented with 10 % fetal bovine serum, 2 % penicillin/streptomycin, and 2 % L-glutamate (GIBCO) and maintained at 37 °C in a humidified 5 % CO_2_/95 % air atmosphere. These cells were either exposed to ethanol for 1, 2, or 4 weeks, or to acetaldehyde for 48 h.

The doses used for ethanol treatment were 0.1 %, 0.3 %, and 1 % by volume (approximate concentrations 17 mM, 51 mM, and 170 mM, respectively). We chose the 0.1 % (17 mM) dose to represent social drinking habits, as 0.1 % is the blood alcohol level constituting legal intoxication in the U.S. [41]. The 0.3 % (51 mM) ethanol dose was used to simulate binge drinking habits, as it is representative of the blood alcohol levels of moderate to heavy drinkers [42]. The 1 % (170 mM) ethanol dose, while potentially lethal in humans, was employed as an upper limit control as it was the highest concentration that was minimally toxic to the oral keratinocytes (Additional file 1: Figure S1). For all ethanol-culture experiments, treatment media was replaced every 24 h with fresh media containing the stated ethanol concentration. The tissue culture plates were also sealed with paraffin film to reduce evaporative loss of ethanol from the media. It has been shown that sealed culture vessels are able to maintain ethanol concentrations over significantly longer incubation periods [43].

The doses used for acetaldehyde treatment were 75 μM, 150 μM, and 300 μM, to represent a range of light to heavy drinking as determined by the saliva concentrations of alcohol consumers [14]. To account for the short evaporation half-life of acetaldehyde, treatments were performed every 4 h and the tissue culture plates were also sealed with paraffin film.

### microRNA expression profiling by qRT-PCR

Total RNA was isolated (Fisher Scientific) from cultured cells after their respective treatments with ethanol or acetaldehyde. cDNA was synthesized using the QuantiMiR^TM^ RT kit (System Biosciences, Mountain View, CA) as per the manufacturer’s instructions. Real-time PCR reaction mixes were created using FastStart Universal SYBR Green Master Mix (Roche Diagnostics), and run on a StepOnePlus^TM^ Real-Time PCR System (Applied Biosystems) using the following program: 50 °C for 2 min, 95 °C for 10 min, 95 °C for 30 s, and 60 °C for 1 min, for 40 cycles. Experiments were analyzed using the ddCt method. U6 primers and a Universal Reverse Primer were used from the QuantiMiR^TM^ RT kit, and custom primers (Eurofins MWG Operon) were ordered using the following sequences: *miR-3178*: 5’-GGGGCGCGGCCGGATCG-3’, *miR-675*: 5’-TGGTGCGGAGAGGGCCCACAGTG-3’, *miR-934*: 5’-TGTCTACTACTGGAGACACTGG-3’, *miR-101*: 5’-CAGTTATCACAGTGCTGATGCT-3’, *miR-1266*: 5’-CCTCAGGGCTGTAGAACAGGGCT-3’, *miR-30a*: 5’-TGTAAACATCCTCGACTGGAAG-3’, *miR-3164*: 5’-TGTGACTTTAAGGGAAATGGCGAA-3’, and *miR-3690*: 5’-ACCTGGACCCAGCGTAGACAAAG-3’.

### microRNA plasmid and siRNA transfections

Expression plasmids for human *miR-30a* and *miR-934* (OriGene Technologies, Rockville, MD) were transiently transfected using Lipofectamine 2000 (Invitrogen, Carlsbad, CA), following the manufacturer’s specifications. The pCMV-MIR empty vector was used as control and transfection efficiency for all three plasmids was monitored using GFP as a reporter.

Knockdown of both miRNAs was performed using Anti-miR^TM^ miRNA inhibitors (Ambion, Austin, TX) that were specific for *miR-30a*-5p and *miR-934*. These inhibitors were also transfected with Lipofectamine 2000.

### qRT-PCR for *BCL-2* gene expression

Total RNA was collected 48 h after transfection with miRNA expression plasmids or inhibitors (Fisher Scientific). cDNA was synthesized using Superscript III Reverse Transcriptase (Invitrogen, Carlsbad, CA) as per the manufacturer’s instructions. Real-time PCR reaction mixes were created using FastStart Universal SYBR Green Master Mix (Roche Diagnostics), and run on a StepOnePlus^TM^ Real-Time PCR System (Applied Biosystems) following the previously described program. Results were analyzed using the ddCt method, with *GAPDH* expression as the endogenous control. Primers were custom ordered (Eurofins MWG Operon, Huntsville, AL) using the following sequences: *GAPDH* forward: 5′-CTTCGCTCTCTGCTCCTCC-3′, reverse: 5′-CAATACGACCAAATCCGTTG −3′. *BCL-2* forward: 5’-CCTGTGGATGACTGAGTACC-3’, reverse: 5’-CCTGTGGATGACTGAGTACC-3’*.*

### Alamar blue cell viability assay

UMSCC-10B and UMSCC-22B cells were plated into a 96-well flat-bottom tissue culture plate (Falcon) at a density of 2,000 cells per well. After a 24 h plating period, these cells were transfected with the miRNA expression plasmids. After a 48 h incubation period, cellular proliferation was analyzed using Alamar blue reagent (ThermoFisher) in accordance with the manufacturer's protocol. All assays were performed in triplicate wells and experiments were individually performed at least twice.

### MTS cell proliferation assay

UMSCC-10B and UMSCC-22B cells were plated into a 96-well flat-bottom tissue culture plate (Falcon) at a density of 5,000 cells per well. After a 24 h plating period, these cells were transfected with the miRNA expression plasmids or inhibitors. For cisplatin sensitivity experiments, the control and transfected cells were subsequently exposed to one of several doses of cisplatin ranging from 0–5 μg/mL. After a 48 h incubation period, cellular proliferation was analyzed using an MTS proliferation assay (Promega) in accordance with the manufacturer's protocol. All assays were performed in triplicate wells and experiments were individually performed at least twice.

### Matrigel invasion assay

Invasion of UMSCC-10B and UMSCC-22B cells was measured using a Matrigel invasion assay (Becton Dickinson, Bedford, MA). 48 h post transfection with either the expression plasmid or inhibitor, cells were trypsinized, and 500 μL of cell suspension (1 × 10^5^ cells/mL) was added in triplicate wells. The lower chamber of the transwell was filled with 750 μl of culture media containing 0.5 % serum as a chemoattractant and allowed to incubate at 37 °C for 24 h. Invading cells on the lower surface that passed through the filter were fixed and stained using crystal violet in gluteraldehyde and photographed. The number of the stained nuclei was counted in a predetermined and consistent section of each well.

### miRNA target gene identification and qRT-PCR

Putative mRNA targets of *miR-30a-5p* and *miR-934* were selected using the TargetScan (v6.2) (http://www.targetscan.org/) and miRDB (v5.0) (http://mirdb.org/miRDB/) target prediction algorithms based on miRNA seed region complementarity in the mRNA 3’UTR.

Total RNA was collected from UMSCC-10B and UMSCC-22B 48 h after transfection with *miR-30a* and *miR-934* inhibitors. cDNA was synthesized using SuperScript III Reverse Transcriptase and qRT-PCRs were performed as previously described, with *GAPDH* serving as endogenous control. Primers for the target genes were custom synthesized (Eurofins MWG Operon) using the following sequences: *GAPDH* forward: 5′-CTTCGCTCTCTGCTCCTCC-3′, reverse: 5′-CAATACGACCAAATCCGTTG −3′. *BNIP3L* forward: 5’- GGACTCGGCTTGTTGTGTTG-3’, reverse: 5’-AGACTGCTCATTTTCCTCGCA-3’. *PRDM1* forward: 5’-TAACACAGACAAAGTGCTGCC-3’, reverse: 5’-CTACCCAGTCCACATTCTCCC-3’. *SEPT7* forward: 5’-ATTCACGCTTATGGTAGTGGG-3’, reverse: 5’-AGCAACTGAACACCACCTTCT-3’. *HIPK2* forward: 5’- CACAGGCTCAAGATGGCAGA-3’, reverse: 5’-GGGATGTTCTTGCTCTGGCT-3’. *HOXA4* forward: 5’-AGAAGATCCATGTCAGCGCC-3’, reverse: 5’-TGTTGGGCAGTTTGTGGTCT-3’. *MLL3* forward: 5’- CTGCCAAAGGAGACTCAGGG-3’, reverse: 5’- GTCCGTTTGCTTCGCTGTTT-3’.

## Additional file

Additional file 1:
**Supplementary data demonstrating effects of short-term ethanol and acetaldehyde exposures at various concentrations on OKF4 and OKF6 cell proliferation and candidate miRNA expression.** **Figure S1.** (A) OKF4 and OKF6 cell proliferation is unaffected by ethanol exposure up to concentrations of 1 % over a 48-h period. (B) OKF4 and OKF6 cell proliferation is unaffected by physiological levels of acetaldehyde exposure over a 24-h period. (C) One-week exposure of OKF4 to ethanol is sufficient to promote upregulation of *miR-934* and *miR-3178*. (PDF 436 kb)
