# Supplementary figures and images for: Alcohol-dysregulated miR-30a and miR-934 in head and neck squamous cell carcinoma
Source: Mol Cancer. 2015 Oct 15;14:181. doi: 10.1186/s12943-015-0452-8 (PMC4608114; doi:10.1186/s12943-015-0452-8)

**A**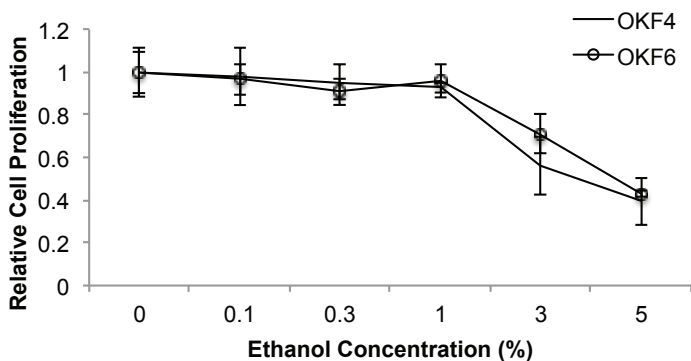**B**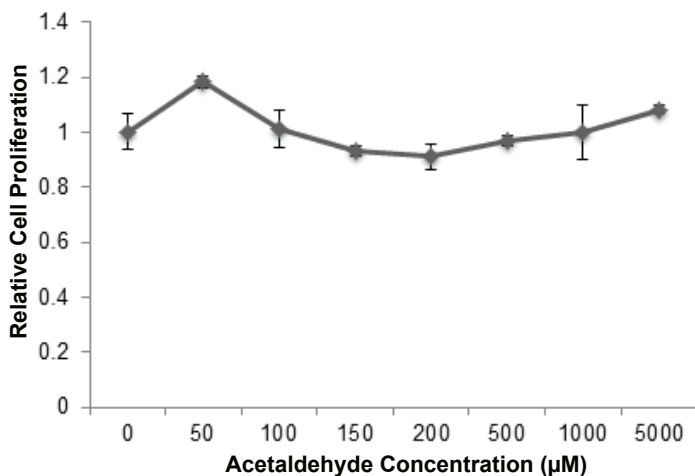**C**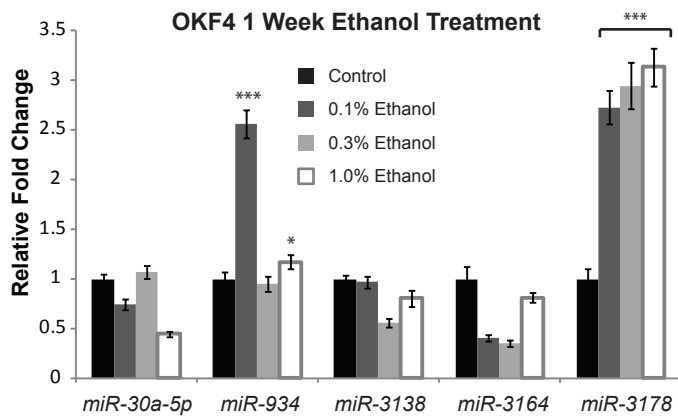

Supplement: Additional file 1: — Supplementary data demonstrating effects of short-term ethanol and acetaldehyde exposures at various concentrations on OKF4 and OKF6 cell proliferation and candidate miRNA expression. Figure S1. (A) OKF4 and OKF6 cell proliferation is unaffected by ethanol exposure up to concentrations of 1 % over a 48-h period. (B) OKF4 and OKF6 cell proliferation is unaffected by physiological levels of acetaldehyde exposure over a 24-h period. (C) One-week exposure of OKF4 to ethanol is sufficient to promote upregulation of miR-934 and miR-3178. (PDF 436 kb) [file 12943_2015_452_MOESM1_ESM.pdf]
